# Voice Analysis for Neurological Disorder Recognition–A Systematic Review and Perspective on Emerging Trends

**DOI:** 10.3389/fdgth.2022.842301

**Published:** 2022-07-07

**Authors:** Pascal Hecker, Nico Steckhan, Florian Eyben, Björn W. Schuller, Bert Arnrich

**Affiliations:** ^1^Digital Health – Connected Healthcare, Hasso Plattner Institute, University of Potsdam, Potsdam, Germany; ^2^audEERING GmbH, Gilching, Germany; ^3^EIHW – Chair of Embedded Intelligence for Health Care and Wellbeing, University of Augsburg, Augsburg, Germany; ^4^GLAM – Group on Language, Audio, & Music, Imperial College London, London, United Kingdom

**Keywords:** neurological disorders, voice, speech, everyday life, multiple modalities, machine learning, disorder recognition

## Abstract

Quantifying neurological disorders from voice is a rapidly growing field of research and holds promise for unobtrusive and large-scale disorder monitoring. The data recording setup and data analysis pipelines are both crucial aspects to effectively obtain relevant information from participants. Therefore, we performed a systematic review to provide a high-level overview of practices across various neurological disorders and highlight emerging trends. PRISMA-based literature searches were conducted through PubMed, Web of Science, and IEEE Xplore to identify publications in which original (i.e., newly recorded) datasets were collected. Disorders of interest were psychiatric as well as neurodegenerative disorders, such as bipolar disorder, depression, and stress, as well as amyotrophic lateral sclerosis amyotrophic lateral sclerosis, Alzheimer's, and Parkinson's disease, and speech impairments (aphasia, dysarthria, and dysphonia). Of the 43 retrieved studies, Parkinson's disease is represented most prominently with 19 discovered datasets. Free speech and read speech tasks are most commonly used across disorders. Besides popular feature extraction toolkits, many studies utilise custom-built feature sets. Correlations of acoustic features with psychiatric and neurodegenerative disorders are presented. In terms of analysis, statistical analysis for significance of individual features is commonly used, as well as predictive modeling approaches, especially with support vector machines and a small number of artificial neural networks. An emerging trend and recommendation for future studies is to collect data in everyday life to facilitate longitudinal data collection and to capture the behavior of participants more naturally. Another emerging trend is to record additional modalities to voice, which can potentially increase analytical performance.

## 1. Introduction

### 1.1. Neurological Disorders and Speech

The burden of neurological disorders on the healthcare system is heavy (1). Neurological disorders manifest themselves with various symptoms at different disease stages. Recognition and diagnosis of most neurological disorders still rely on clinical examinations, mostly upon the manifestation of prominent symptoms. With modern machine learning approaches, researchers have attempted to quantify neurological disorders through various modalities from unobtrusive sensors to gain a longitudinal and holistic picture of the individual patient and course of disease (2). Speech, in particular, is a promising modality, since its production is shown to be very susceptible to slight perturbations caused by those disorders (3). Furthermore, voice recordings are unobtrusive and readily available through the widespread usage of smartphones and other smart devices (4).

To record voice data in a clinical setting, the principle approach is to access a patient cohort and compare it with a representative healthy control cohort. An experimental protocol is developed, which includes a medical assessment to quantify the disorder as well as the recording of voice samples according to clearly defined speech elicitation tasks. The medical assessment provides a ‘ground truth' description of the disease status, and the voice recordings are then used to indirectly infer that disease status.

Existing studies have regarded a multitude of neurological disorders, which were reported to have a measurable impact on voice. Those can be loosely grouped, for the scope of this review, into psychiatric disorders, neurodegenerative disorders and speech impairments. Psychiatric disorders encompass depression (3), anxiety, obsessive-compulsive disorder (OCD), post-traumatic stress disorder (PTSD) (5), schizophrenia (6), and, to a certain extent, stress (7). Neurodegenerative disorders include disorders leading to cognitive decline, such as Alzheimer's disease (AD) and mild cognitive impairment (MCI) (8, 9), as well as a broader range of disorders that do not primarily affect cognition, such as amyotrophic lateral sclerosis (ALS) (10), multiple sclerosis (MS) (11), and Parkinson's disease (PD) (12). Lastly, there are several disorders, which affect speech production itself, such as aphasia, dysarthria, and dysphonia. Aphasia is the inability to comprehend or formulate language, dysarthria emerges when muscle coordination for speech production is impaired and dysphonia is when voice is hoarse due to problems with the larynx.

### 1.2. Data Processing Pipeline

#### 1.2.1. Speech Tasks

The human voice can be produced in various ways, such as reading text, singing or laughing. Recommendations for the technical details on how data for the acoustic assessment of voice production in a clinical setup should be recorded, are provided by Patel et al. (13). These guidelines are compiled by an expert panel from the American Speech-Language-Hearing Association (ASHA), and we strongly recommend consulting these suggestions before setting up novel data collection efforts.

In research settings, participants are asked to produce specific vocalisations, which elicit distinct information for comparable analyses. Those speech tasks, which provide the basis for voice-based disorder quantification, can be grouped into certain categories for the scope of this review. Participants can be asked to produce a sustained phonation of a phone, typically the vowel /a/. Diadochokinesis is the ability to produce antagonistic movements in quick succession, these are typically rapid syllable successions in the case of speech tasks, such as pa-ta-ka. Read speech categorizes tasks, in which written material is provided to be read out aloud. Those materials can be customized for a specific research question or standardised text passages, for example ‘the north wind and the sun,' which is constructed to contain every phone in the English language. Free speech encompasses tasks, which do at most provide an initial association point, but then require the participant to associate or behave freely. Examples are clinical interviews between a physician and a patient or a ‘picture description task,' in which the patient is asked to describe a picture in their own words.

#### 1.2.2. Feature Extraction

With the obtained data at hand, data analysis is performed next. The typical data analysis pipeline consists of preprocessing the collected data and then applying analytical methods to obtain quantitative insights. The very first step here is to enhance the quality of the raw audio signal by applying, amongst others, denoising and dereverberation. For data preprocessing, audio recordings are often filtered for segments containing speech through voice activity detection (VAD). If multiple speakers are present in one recording, speaker diarisation can be applied to try to separate voice segments, for example, from the patient and a doctor in a clinical interview setting. To perform linguistic analysis, recent advances in automated speech recognition (ASR) enable automatic transcription of the content. With transcriptions, analysis can include, for example, aspects of the semantic structure of the recorded speech [e.g., as done by Tóth et al. (14)].

To make the raw audio signal accessible for automated analysis, statistical derivatives of the signal, namely, features, are extracted. To quantify voice, several features stem from the acoustic aspects of the speech signal that account for the structure of the vocal production system. Prominent and commonly used acoustic feature sets in the community are the expert-knowledge driven Geneva Minimalistic Acoustic Parameter Set [GeMAPS, Eyben et al. (15)] on one hand and the large-scale, general-purpose driven Computational Paralinguistics Challenge [ComParE, Weninger et al. (16)] feature set. Further, there are features, which are tailored for disease-specific vocal dynamics [e.g., (8) on AD]. Low et al. (17) provide a comprehensive overview of the commonly used acoustic features derived from speech in neurological disorder quantification. They regard the GeMAPS features and provide a glossary on the regarded features [based on Cummins et al. (3) and Horwitz et al. (18)], to which we refer the interested reader.

Recent additions to those ‘traditional' acoustic features were introduced at ComParE 2018 and 2019 (19, 20), and are based on representations of the audio signal found through deep neuronal networks (see 1.2.3 Analysis), as well as a high-level summary of speech segments through the Bag-of-Audio-Words (BoAWs) approach. There are out-of-the-box toolkits to extract features, most prominently Praat (21), openSMILE (22), and VOICEBOX. BoAWs can be extracted using the openXBOW framework (23), and learnt representations of the speech signal can be extracted with the DeepSpectrum (24) and AuDeep (25, 26) toolkits. Nonetheless, it is not uncommon to write custom code to perform feature extraction.

#### 1.2.3. Analysis

After preprocessing and feature extraction, data analysis is performed. There are two general approaches for data analysis: statistical analysis and predictive modeling.

For statistical analysis, extracted features are tested with various statistical means to find significant correlations of individual features for the tested conditions, which then express changes in vocal characteristics. The sum of those identified correlating features can ideally serve as general and reliable indicators for different disorders, and are occasionally referred to as ‘vocal biomarkers.'

In predictive modelling, on the other hand, machine learning approaches are used to try and build statistical models, which can discriminate between different categories or a general scale, relevant for the regarded disease at hand. Common machine learning models employed for categorical classification are, support vector machines (SVM), the k-nearest neighbors algorithm (k-NN), decision trees (DT), random forests (RF), Gaussian Mixture Models (GMMs), and Hidden Markov Models (HMM). If values from continuous scales are to be predicted, regression models, such as linear regression, logistic regression, support vector regression, and regression trees can be utilised.

If sufficient data is available, artificial neural networks (ANN) can be employed as well, which promise a high performance on large data sets. For ANNs, organizational architectures of neuronal networks inspired by the dynamics in the human brain, are constructed for specific tasks in specific domains. In the field of disease recognition from voice, convolutional neural networks (CNNs) and long short-term memory (LSTM) networks are commonly used, see Cummins et al. (27) for a review of recent developments and examples in the field. Foremost, CNNs learn feature representations of input spectrograms of the audio signal or directly from the raw audio waveform. They either contain architectural elements to perform a classification decision right within the network architecture or other predictive modeling approaches are employed based on those feature representations.

With ComParE 2018 and 2019 (19, 20), learnt deep representations are used as additional baseline feature sets. With the DeepSpectrum toolkit, CNNs pre-trained for image recognition tasks, are used to extract abstract representations of spectrograms from the raw audio signal. auDeep first uses spectrograms from the input audio signal to train encoder-decoder networks without providing class labels (sequence-to-sequence autoencoder), specific to the data at hand. The outputs of the trained encoder can then be used to output features in the form of abstract representations based on the spectrograms of the input signal.

### 1.3. Related Work

Previous reviews in the field have summarized the state of voice analysis for individual disorders and a few reviews outlined the state of research across several neurological disorders. One prominent systematic review was performed by Low et al. (17), in which they regarded a variety of psychiatric disorders (depression, PTSD, OCD, bulimia, anorexia, schizophrenia, hypomania, and anxiety). Therein, they synthesised which acoustic features are prominently changed in voice in each disorder. They further provided an overview of recent developments and guidelines for data collection. Another review was performed by Voleti et al. (28), which regarded neurological thought disorders (such as AD, schizophrenia, etc.) and created a taxonomy for speech and language features used.

However, the scope of the review of Low et al. (17) was limited to psychiatric disorders and Voleti et al. (28) did not perform a systematic literature search. In this context, a comprehensive review that provides a broad overview of the field of neurological disorder recognition from voice is needed. Therefore, we extended to the scope of Low et al. (17) by also including the neurodegenerative disorders ALS, AD, MCI, MS, and PD. Further, we adopted a reproducible, systematic approach by querying bibliographic databases.

### 1.4. Scope of the Review

The aim of this review is to provide a general overview of the field of neurological disorder recognition from voice. The main contribution is to survey how voice data is commonly collected across psychiatric and neurodegenerative disorders, how data is frequently analysed, and to highlight emerging trends. The novel insights from this review will be helpful when setting up future data collection efforts.

**Figure 1:**
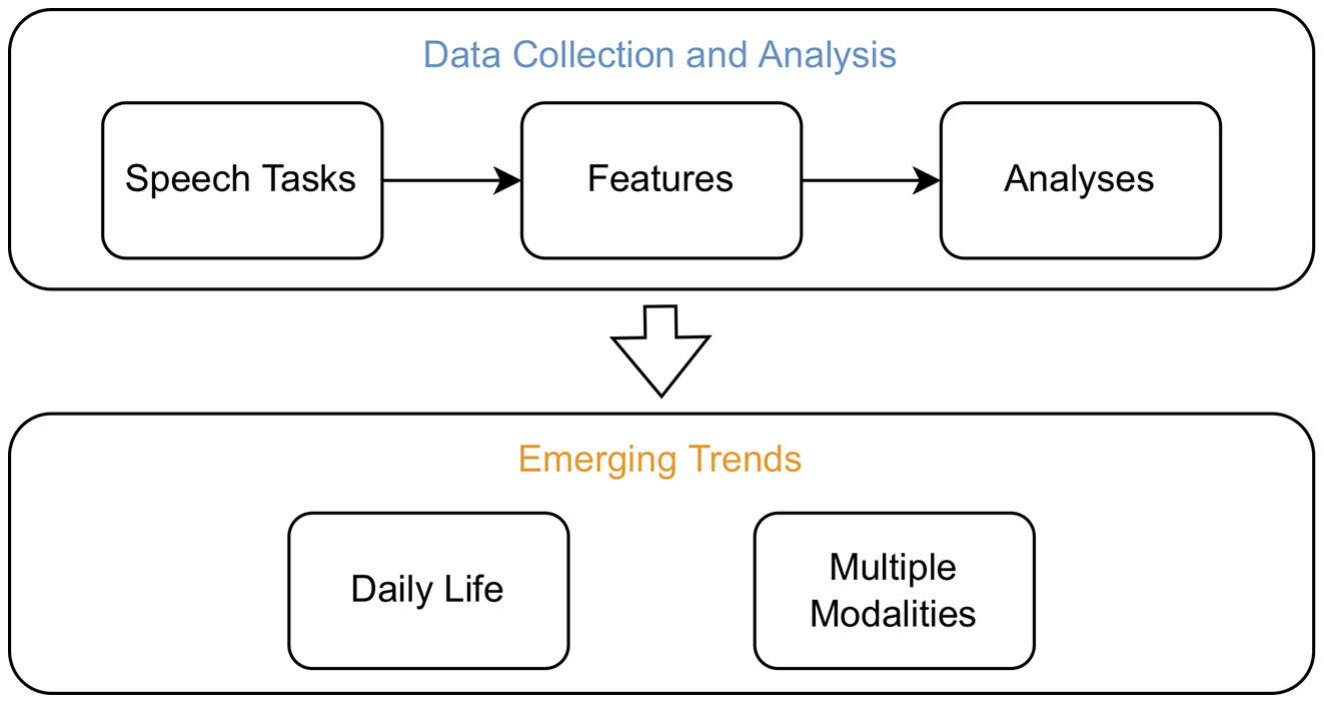
Overview of the topics addressed within this review. Studies which recorded original datasets to assess neurological disorders from voice were screened. From those, information was extracted to assess which speech tasks are commonly employed, which methods are frequently used to extract features, and which analysis methods are prevalent. In addition, significantly correlating acoustic features in common psychiatric and neurodegenerative disorders were summarised. The emerging trends to record data in daily life and from multiple modalities were uncovered by screening for particularities in the recording setup of the retained studies.

We do this by searching for publications on original datasets. From these retrieved publications, we extract information on the study setup, the speech tasks utilised, the analysis methods used, and particularities in the voice recording setup (to uncover emerging trends). Furthermore, we provide an overview of significantly correlating acoustic features in common psychiatric and neurodegenerative disorders by extending the work of Low et al. (17). Figure 1 presents an overview of these outlined topics addressed within this review.

## 2. Materials and Methods

This systematic review was conducted according to the Preferred Reporting Items for Systematic Reviews and Meta-Analysis (PRISMA) guidelines (29).

### 2.1. Literature Screening

#### 2.1.1. Information Sources/Identification

The following electronic databases were searched for relevant articles: PubMed, Web of Science (Web of Science Core Collection, version 5.35), and IEEE Xplore. Those databases were queried in August 2020 with the following search term: ((speech OR voice) AND (dataset OR ‘data set') AND <disorder specification>). In place of <disorder specification>, a search term for each regarded disorder was inserted:

- (‘mental health' OR psychiatry OR psychiatric OR ‘affective disorder' OR ‘psychological disorder' OR ‘mental illness')
- (Anxiety)
- (Depress*)
- (Stress)
- (‘Acute stress reaction')
- (‘Obsessive-compulsive disorder' OR OCD)
- (‘Post-traumatic stress disorder' OR PTSD)
- (Schizophrenia)
- (Hypomania)
- (Bulimia)
- (Anorexia)
- (Alzheimer*)
- (Dementia)
- (‘Cognitive impairment*')
- (‘Multiple sclerosis')
- (Parkinson*)
- (Aphasia).

The disorders to be regarded were primarily based on work from other reviews on individual and multiple disorders. The aim was to cover psychiatric as well as prominent neurodegenerative disorders, stress as well as speech impairments such as aphasia. No restriction on the date of publication was imposed.

Google Scholar is an ambivalent source for systematic literature reviews. On one hand, it covers a broad range of publications, especially those in conference proceedings, but on the other hand, it is crawler-based instead of bibliographic and more focused on exploitative instead of systematic search behavior and does not allow bulk downloads of the returned results (30). Therefore, we decided not to use Google Scholar for the systematic search here but can recommend it as well as explicit dataset search engines such as Google Dataset Search to the interested reader to explore individual disorders and aspects of the field.

#### 2.1.2. Screening

Only articles published in English language were considered. After duplicate removal, the first author (P.H.) screened the title and abstract of all records. The focus was to include studies, which report a newly recorded (‘original') dataset, and whose research was primarily based on voice and speech. Emphasis was put on studies, which regraded acoustic features (omitting purely linguistic analyses to keep the scope manageable). Studies had to focus on the above-mentioned disorders and include recording voice data from patients. The exclusion criteria for screening were: (a) publications that used existing datasets (i.e., did not record data themselves), (b) publications that were not focused on the above-mentioned neurological and psychiatric disorders, studies involving children, publications, which focused on qualitative or quantitative interview analyzes as well as literature reviews. 203 duplicates were removed with the ‘check for duplicates' function in the reference manager Mendeley Desktop (version 1.19.6, Elsevier, Amsterdam, Netherlands); the other bibliography organization of this literature review was done in Zotero (version 5.0.90, Corporation for Digital Scholarship, Vienna, Virginia, USA).

### 2.2. Data Extraction

Data extraction was performed by P.H. with assistance of N.S. Our approach was to extract a wealth of information to assess common practices and to identify emerging trends in the field later on. Data to be extracted consisted of information on (a) the study setup (number of patients and patient assessment), (b) the voice recording setup (additional modalities, recording conditions: in everyday life or laboratory), (c) the speech tasks (elicitation protocols) used in the study (elicitation material used, if applicable: performance comparison), and (d) analysis methods employed (features extracted, analysis methods used: statistical and predictive modeling and validation schemes).

In published studies, the focus is often put on analysis and it is not clearly stated in the title or abstract, whether original data was recorded or an existing dataset was used. The search term (‘dataset' or ‘data set') in this systematic review was introduced to search for original datasets However, some original studies might not have been covered. Therefore, we conducted an additional systematic search for literature reviews, which are focused on acoustic analysis of individual disorders and synthesized their identified features.

### 2.3. Acoustic Features

The aspect of which acoustic features are found to correlate with which neurological disorder was addressed prominently by Low et al. for psychiatric disorders (17). In the broader scope of this review, we aimed to extend their synthesis to also incorporate acoustic features of the neurodegenerative disorders addressed in this review.

Several recent reviews summarized significantly correlating acoustic features in individual neurodegenerative disorders, and we systematically screened an electronic database to retrieve those. We queried Web of Science and used their ‘refine' function to retain only review articles published from 2015 on. The search terms to retrieve reviews were:

- TS=((ALS) AND (speech OR voice) AND (analysis))
- TS=((Alzheimer*) AND (speech OR voice) AND (analysis))
- TS=((Multiple sclerosis OR MS) AND (speech OR voice) AND (analysis))
- TS=((Parkinson*) AND (speech OR voice) AND (analysis))
- TS=((stress) AND (speech OR voice) AND (analysis)).

Title and abstract were screened and full-text articles were retrieved for the matching candidates. Reviews that provided syntheses in which publications were explicitly listed that found correlating acoustic features with the respective disorder, were retained.

With the publicly available source code^1^ and permission provided by Low et al. (17), we extended their synthesis of Figure 3 by adding data of the studies listed in the found reviews. Studies identifying a significant positive correlation received a score of 1, studies finding a significant negative correlation received a score of –1 and non-significant or contradictory studies were scored with 0. Only the most comprehensive review on each disorder (clearly stating the studies found with correlating acoustic features) was used so to cover a comparable number of studies. Reviews used to extract data for extending the figure were the following (9–12). The code to extend the figure of Low et al. (17), and to plot all figures from this review, can be found at GitHub^2^. The aspects of stress and speech impairments were omitted from that overview to fully focus on neurodegenerative disorders.

Furthermore, stress and speech impairments were found to be very heterogeneous. Different manifestations of stress were described by Van Puyvelde et al. (7) for physical, delirious, emotional, and cognitive load and they presented an own model for Voice and Effort (MoVE) to characterize those interactions with voice. Speech impairments such as aphasia, dysarthria, and dysphonia amongst others, stem from general dysfunctions of the speech production systems, and for example, dysarthria can be the consequence of stroke as well as MS.

## 3. Results

**Figure 2:**
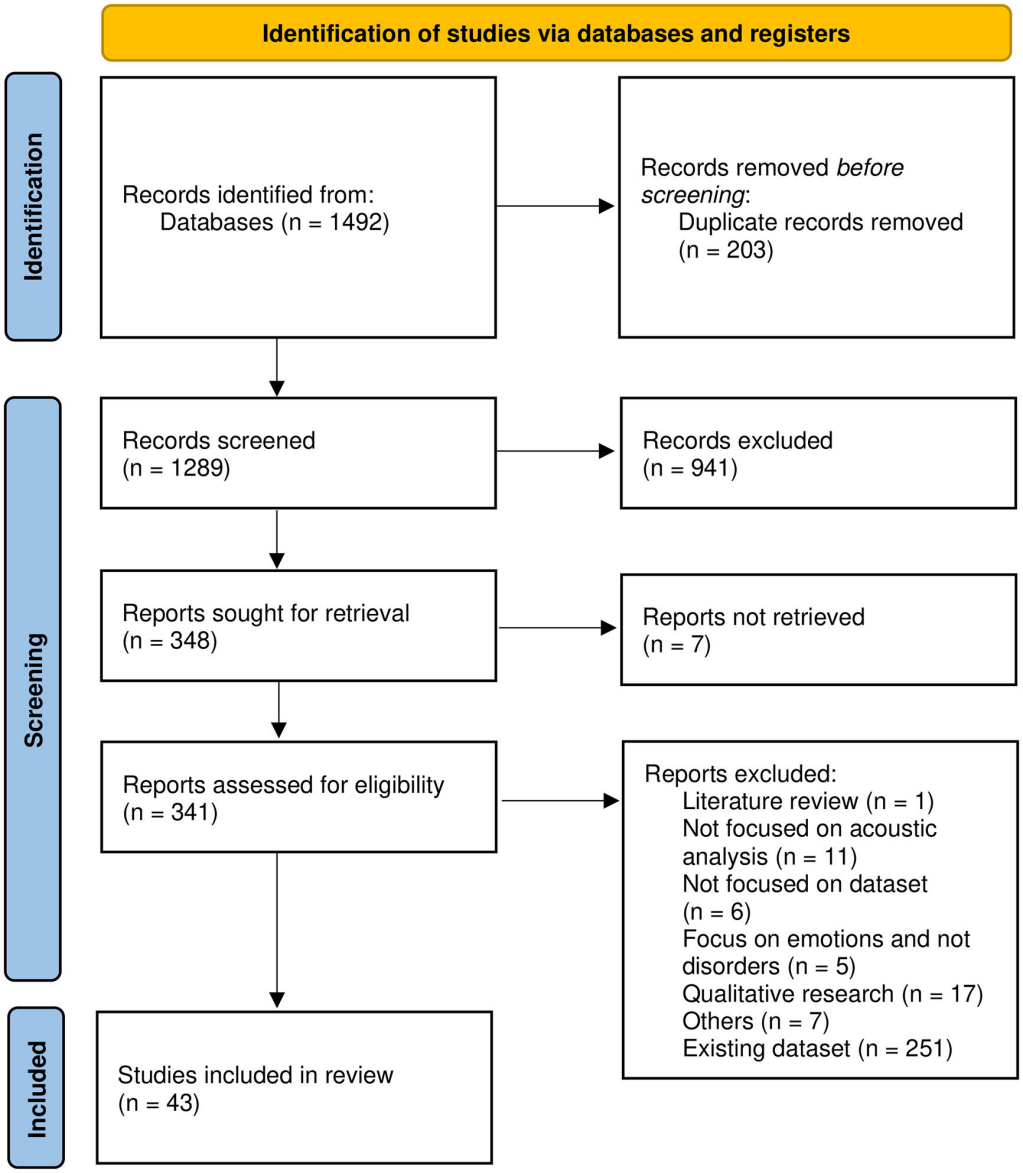
PRISMA flow chart for study selection.

The PRISMA flow diagram is depicted in Figure 2 and shows the study selection process.

The search terms described in 2.1.1 were used to retrieve 1,492 publications and ultimately, 43 studies were included.

After obtaining the final included studies, we noticed that the disorders described in those studies fell into slightly different categories than searched for in the search terms. The categories that started to emerge after data extraction were the following: the neurodegenerative disorders ALS, AD, and PD, the psychiatric disorders bipolar disorder, depression and, to some extend, stress as well as the group of speech impairments, such as aphasia, dysarthria, and dysphonia. Our results and the discussion are therefore based on those categories.

Table 1 presents the number of studies found for each disorder and summary statistics on the number of participants (patients and controls) for all studies of each disorder. Most studies describing original datasets were included for PD followed by stress. PD also has on average most patients included, while for datasets on stress, usually no patients but only healthy participants are recruited.

**Table 1:** Overview of the included studies reporting on original (newly recorded) datasets from neurological disorders to provide a survey over emerging trends in the field.

**Disorder**	**# studies**	**Patients**	**Controls**
		**Median (range)**	**Median (range)**
Parkinson's	20	36 (3–1,513)	20 (8–64)
Stress	6	-	44 (4–60)
Depression	5	92 (12–224)	61 (12–397)
Speech impairments	4	12 (8–21)	13 (8–21)
Alzheimer's	3	82 (71–214)	93 (82–268)
ALS	3	13 (11–25)	12 (11–13)
Bipolar	2	31 (10–51)	9 (9)

### 3.1. Speech Tasks

**Figure 3:**
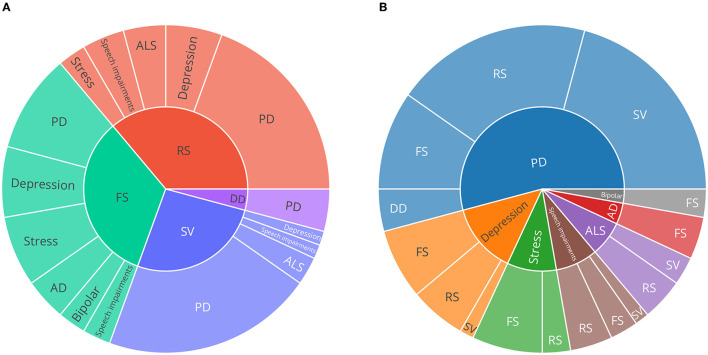
Sunburst charts describing the proportion of speech tasks and neurological disorders. **(A)** speech task categories on the inner circle, **(B)** disorders on the inner circle. Only publications describing original (newly recorded) datasets were considered to provide an overview of emerging trends in the field. PD, Parkinson's disease; AD, Alzheimer's disease; ALS, amyotrophic lateral sclerosis; SV, sustained vowels; DD, diadochokinesis; RS, read speech; FS, free speech.

Figure 3 is a synthesis of the included studies and provides an overview of the proportion of how often each speech task was recorded for each disorder. To provide an overview of the proportion of speech tasks represented in general, dependent on disorder, Figure 3B is an inverse view on the data of Figure 3A. Here, it is noticeable that speech tasks eliciting free speech (FS) are used most frequently in the included studies. Furthermore, that speech task category was used in all disorders analysed, except for ALS.

Since studies could employ multiple speech tasks, the number of speech tasks may differ from the number of original datasets (Table 1). Roughly half of the speech tasks described were utilised in datasets collected from PD patients, stress was represented second often.

In comparison to the other speech tasks regarded in this review, free speech and read speech tasks are less strictly defined. Nevertheless, several typical setups could be identified. Common setups for free speech tasks include (clinical) interview situations (31–37), acted interactions (38–40), picture description (41–44), letting participants talk about a specific question or topic (45–47), or even smartphone conversations (48, 49), as well as specific memory and association tasks suitable for quantifying AD (44). Read speech includes standardised (36, 42, 47, 50) and custom (51–60) sentences or text passages, such as ‘the north wind and the sun' (46, 61), ‘the rainbow passage' (62, 63), and other passages (64, 65) as well as disease specific tasks, such as constructed sentences with emotionally evoking words for depression quantification (31, 66). Especially in PD, utilising sustained phonation of the vowel /a/ appear to be popular [e.g., (60, 67–71)]. The most specific speech task used was diadochokinesis (DD), which was only used in datasets concerned with PD [e.g., (67)].

Data underlying Figures 3A,B, resulting from data extraction, are included in Supplementary Tables S1, S2.

### 3.2. Feature Extraction

**Figure 4:**
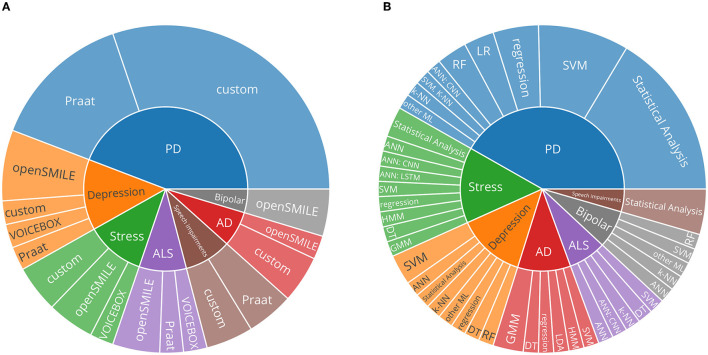
**(A)** Sunburst chart describing the proportion of feature extraction toolkits used and neurological disorders. ‘Custom' describes studies which did not mention the toolkit used or which utilised custom methods to extract features. **(B)** Sunburst chart describing the proportion of predictive modeling approaches used and neurological disorders. Categorical classification: SVM, support vector machines; k-NN, k-nearest neighbors algorithm; DT, decision trees; RF, random forests; GMM, Gaussian Mixture Models; HMM, Hidden Markov Models; Regression models: LR, linear regression; regression: other regression methods. ANNs, Artificial neural networks; CNN, convolutional neural networks; LSTM, long short-term memory networks; PD, Parkinson's disease; AD, Alzheimer's disease; ALS, amyotrophic lateral sclerosis.

Figure 4A presents a synthesis of the feature extraction toolkits used. Praat, openSMILE, and VOICEBOX emerged as commonly used out-of-the-box toolkits for feature extraction. Roughly half of the included studies used custom code or did not specify the toolkit used.

### 3.3. Analysis

#### 3.3.1. Statistical Analysis

Figure 4B aggregates broad categories of analysis methods. Statistical analyses, where individual features are tested for significance, are relatively frequently used.

**Figure 5:**
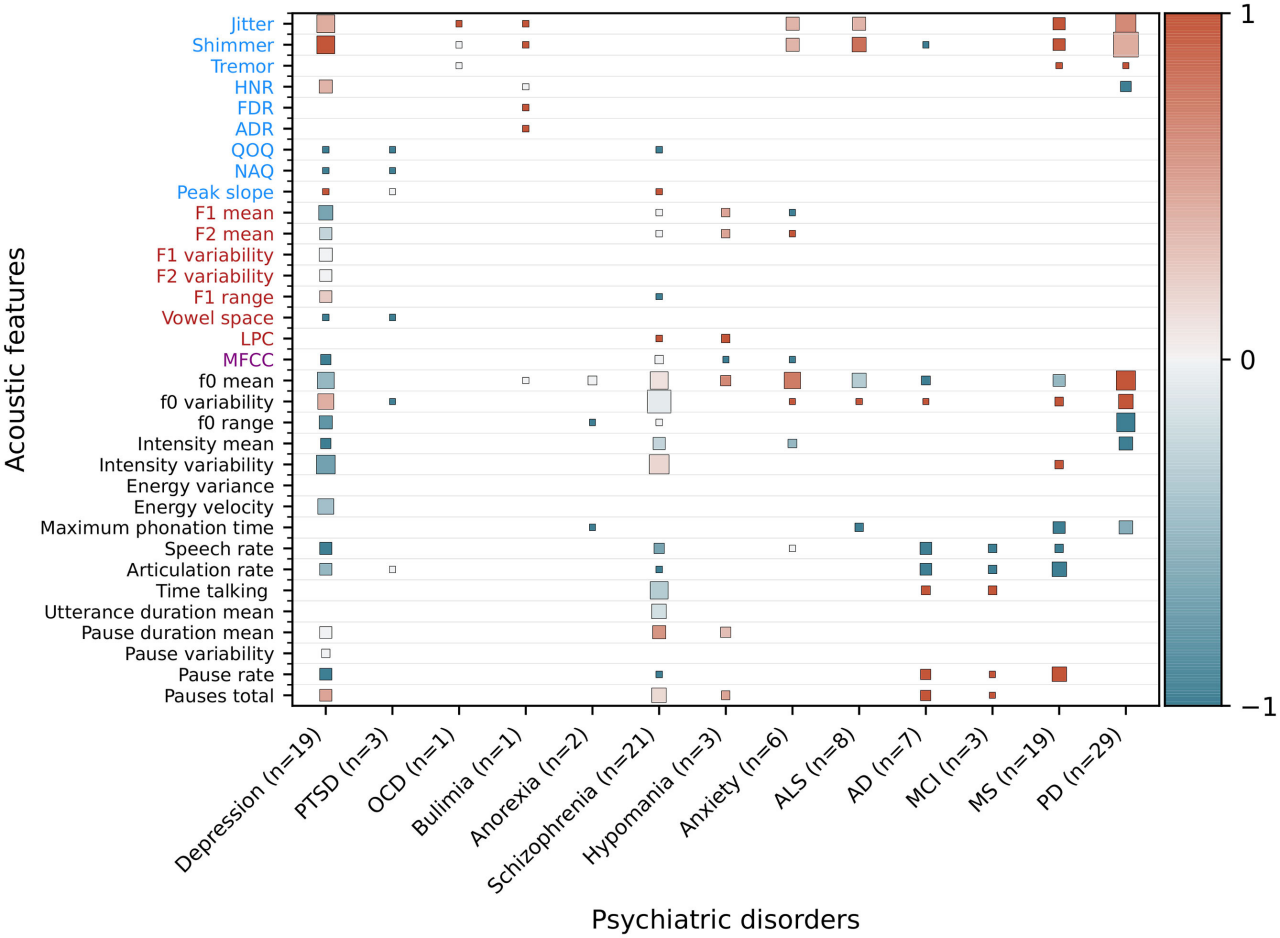
Extended heatmap based on Figure 3 from Low et al. (17). In addition to psychiatric disorders, significantly correlating features from neurodegenerative disorders were extracted and added based on recent reviews of the respective disorders. Features that are significantly higher in a psychiatric population than healthy controls or that correlate positively with the severity of a disorder receive a score of 1 (red), features that are lower or correlate negatively receive a score of –1 (blue), and non-significant or contradicting findings receive a score of 0 (gray). The mean is computed for features with multiple results. The cell size is weighted by the number of studies. Features not studied in a disorder are blank. Additionally, the number of studies (n), of which the correlating features are extracted, is given for each disorder. OCD, obsessive-compulsive disorder; PTSD, post-traumatic stress disorder; ALS, amyotrophic lateral sclerosis; ALS, amyotrophic lateral sclerosis; AD, Alzheimer's disease; MCI, mild cognitive impairment; MS, multiple sclerosis; PD, Parkinson's disease.

Figure 5 is an extended version of the synthesis created by Low et al. (17). Acoustic feature categorisation is based on Eyben et al. (15). Each cell represents a summary of the studies with statistical tests performed for the respective feature. The more studies were found for a respective feature, the larger the cell. The found correlation of each study determines the shading: if a feature correlates positively with the disorder, the cell is shaded red. In case of a negative correlation, the cell is shaded blue and if non-significant findings are presented, the shading is gray. The final shading of a cell is determined by accounting for all correlations for all reported studies: the more intense, the more unanimous the findings across all studies and the less intense, the less unanimous are the aggregated studies. For each of the added neurodegenerative conditions, a review was systematically identified, which synthesized several studies which reported correlations of acoustic features with the respective condition. The review used to extract studies for ALS was (10), the one for AD and MCI was (9), the one for MS was (11), and the one for PD was (12).

#### 3.3.2. Predictive Modeling

The predictive modeling approaches pursued by the retrieved studies are presented in Figure 4B. Classical (non-neural-network-based) approaches are in the majority. Of those approaches, support vector machines followed by regression approaches, are most prominent. General artificial neural networks (ANNs) and convolutional neural networks (CNNs) are most widespread in the included studies. Neural networks can consume the raw audio signal in various ways. The introduced learnt representations with DeepSpectrum and AuDeep were used in Baird et al. (61), and AuDeep achieved the best results. Often, features based on the mel-frequency cepstral coefficients (MFCCs) are used as input to the studies that employ deep learning approaches. MFCCs, simplified, aim to represent a spectrum based on how speech is perceived by human hearing. Mendiratta et al. (46), Khorram et al. (48), and An et al. (56) use MFCCs to represent the speech signal for their deep learning approaches. In addition, Khorram et al. (48), Baird et al. (61), An et al. (56), and Prince et al. (72) provide hand-crafted feature sets to the neural network, for example, Khorram et al. (48) used eGeMAPS features as input for a ANN.

Table 2 shows datasets, in which further modalities in addition to audio were recorded. Only included datasets for ALS, PD and stress recorded multiple modalities.

**Table 2:** Included studies with original datasets, in which multiple modalities were recorded.

	**Year**	**Condition**	**Additional modalities**
Garcia-Gancedo et al. (65)	2019	ALS	Physical activity, heart rate variability (HRV)
An et al. (56)	2018	ALS	Articulatory movement data
Wang et al. (53)	2016	ALS	Articulatory movement data
Prince et al. (72)	2019	Parkinson's	Sensor data: Finger tapping, walking, memory task
Barnish et al. (36)	2017	Parkinson's	Video, Respiratory Sinus Arrhythmia (RSA) and Heart Rate (HR)
Gratch et al. (32)	2014	Depression	Videos
Baird et al. (61)	2019	Stress	Biosignals: Blood volume pulse (BVP), Skin conductance (SC)
Lefter et al. (38)	2014	Stress

Table 3 presents datasets, in which data was collected outside a controlled laboratory setup (‘in everyday life'). Recordings here were most prominently done via user smartphones or web forms.

**Table 3:** Included studies with original datasets, in which data was collected outside a traditional laboratory setup: in everyday life.

	**Year**	**# Subjects**	**Condition**	**Recording condition**
Khorram et al. (48)	2018	60	Bipolar disorder	Conversations during daily smartphone usage
Maxhuni et al. (49)	2016	10	Bipolar disorder	Smartphone recorded constantly in the background
Zhang et al. (45)	2020	222	Depression	Web forms
Prince et al. (72)	2015	1,513	Parkinson's	User smartphones
Dubey et al. (55)	2015		Parkinson's	Smartwatch in group session for vocal exercises
Palacios-Alonso et al. (40)	2019	32	Stress	Smartphone
Garcia-Gancedo et al. (65)	2019	25	ALS	Home monitoring and clinical site visits for sensor data recording; audio only collected at clinical site

## 4. Discussion

In this review, we systematically screened for publications, in which voice data for various neurological disorders were recorded. Syntheses of included studies provide a high-level overview of different disorders and insights into emerging trends in the field. Previous work was extended to provide an overview of which features are correlated with changes in voice in psychiatric and neurodegenerative disorders.

The respective subsections in the discussion aim to provide valuable guidance when performing such data collection. We cover the aspects of which speech tasks are frequently used, which confounders might be encountered, which feature extraction toolkits are available, which analysis methods are common, and which validation procedures should be employed.

### 4.1. Neurological Conditions and Speech

As presented in Table 1, systematic literature screening returned the most original datasets for PD. Research done in this domain was one of the earliest approaches in the whole field of speech analysis for disease recognition (73) and therefore, the high aggregation of datasets could be reasonable. ALS and bipolar disorder, on the other hand, appear to be relatively under-explored research areas in terms of datasets published.

#### 4.1.1. Speech Tasks

When regarding the numbers of speech tasks used for different disorders as presented in Figure 3, it appears that the free speech task category is most commonly used in existing datasets, closely followed by read speech with only one dataset less. Both task categories show broad heterogeneity and can be divided into individual subcategories. In essence, however, free speech tasks aim at capturing ‘naturally flowing' speech, in which especially hesitations and pauses can be valuable disease indications, for example, when regarding AD or MCI (9). A very standardised approach used across multiple disorders appears to be the picture description task, utilised in PD (41), stress (42), and AD (43, 44). Recently, Slegers and Filiou (74) reviewed several studies that employ picture description tasks to describe their potential in clinical practice to assess AD. Similarly, Mueller et al. (75) assessed how picture description tasks can be used in diagnosing AD and even potentially already in MCI. Speech tasks prompting read speech cover a wide range of the participant's language (in contrast to e.g., the task of the constrained sustained pronunciation of vowels), while still having a fixed body of text that is consistent for all participants.

A few publications ced the performances of different speech tasks used in the same dataset. This can provide valuable insights into which tasks appear to cover the best information on a disorder status in an actual recording setup. However, only 6 of the included publications provide those analyses, therefore, unfortunately, these reports can be only regarded as anecdotal. Sakar et al. (57) and Karan et al. (51) each report in their analysis on PD that performance on sustained phonations of vowels performed better than read speech. Interestingly, (59) recorded Czech speaking participants with and without PD and regarded a neutral and a word-stress-modified reading passage and found that the passage with word stress modifications performed better. Further, they achieved their best performance with a free speech task, in which participants had to recite a poem from memory. Alghowinem et al. (31), Liu et al. (37), and Zhang et al. (45) reported that tasks using free speech performed better than sustained vowels and read speech for depression, respectively. A recent study assessed differences in performance of various speech tasks eliciting connected speech in patients with early AD and MCI. That study, therefore, offers some practical consideration for which particular free speech task might be best suitable for these conditions (76). Analysing the performance of speech tasks is valuable for the community, since choosing the best performing speech task can reduce time effort and burden imposed on the patient in a clinical as well as in an everyday-life setup.

#### 4.1.2. Confounders

In their review (section 4.2), Low et al. (17) portray several relevant confounding factors, which should be considered and avoided during data collection. Regarding rather symptoms and problems and not only disorder rating scales promises to provide a more fine-grained view of a patient and account for disorders, in which more heterogeneous symptoms are present (77). A central aspect that needs to be controlled for in voice analysis, are confounding factors that influence voice production. Commonly assessed factors are, for example, age, sex, and native language, less common are comorbidities, race, education, height, weight, and dialect. Especially medication is not frequently reported but plays a crucial role since its side effects might influence speech production.

### 4.2. Data Processing Pipeline

#### 4.2.1. Feature Extraction Toolkits

Regarding the toolkits used for feature extraction, as portrayed in Figure 4A, of all studies actually extracting features, almost half used custom methods. In particular, in the field of PD, datasets are described, which validate and explore the impact of Lee Silverman Voice Treatment (LSVT) (78) to mitigate voice-based impairments due to PD. Success in that treatment routine is measured in increased vocal intensity [e.g., (63, 79)], and therefore in those studies, features are very specific and focused only on that outcome. As pointed out by Low et al. (17), standardising feature extraction yields the benefit of better comparability across studies, but specific approaches in which anatomically informed and manually constructed features can reflect an aspect of a disorder, which might not be covered by standardised feature sets, can be valuable as well. Within the scope of this review, relevant feature extraction toolkits were presented. Studies using custom methods are hard to quantify systematically since the performance obtained on one dataset might not transfer well to another dataset. Further, it is worth emphasising that, since studies included in this review are limited to original datasets, the actual usage in all analytical studies might vary.

#### 4.2.2. Features Correlating With Neurodegenerative Conditions

We extended the figure of the synthesis of significantly correlating features for neurological disorders in Low et al. (17) by adding the neurodegenerative conditions ALS, AD and MCI, MS, and PD (Figure 5). Findings regarding the disorder-related features are summarized as the following:

*Amyotrophic lateral sclerosis*: Chiaramonte and Bonfiglio (10) conducted a meta-analysis and found that jitter and shimmer correlate positively, and maximum phonation time (MPT) correlates negatively, significantly with progression of bulbar ALS. The predominantly initial spinal type of ALS, characterised by muscle weakening, usually transitions to show some bulbar involvement at a later stage, at which speech impairments are surfacing. No significant correlations between F0 mean and F0 variability were observed in the meta-analysis.

*Alzheimer's disease and mild cognitive impairment*: Martínez-Nicolás et al. (9) systematically reviewed altered acoustic features in patients with AD and MCI. Decreased speech and articulation ratio, as well as an increased number of pauses, are characteristic for the early stages of AD. Fewer studies are concerned with MCI, but increased pause duration and longer speech and phonation time are reported. Language impairments are already present in the prodromal (pre-symptomatic) stage and the challenge of the field is to distinguish cognitive impairments due to age from the onset of AD.

*Multiple sclerosis:* Noffs et al. (11) systematically screened for studies describing speech impairments in MS and found, for acoustic analyses, that a slowing in tongue movement causes a lower speech and articulation rate. Further, glottal inefficiency causes increased jitter and shimmer, and intensity variability and symptoms are expected to worsen upon disease progression.

*Parkinson's disorder*: Chiaramonte and Bonfiglio (12) conducted a meta-analysis and concluded that jitter, shimmer and F0 variability are significantly increased in patients with PD. Increased F0 variability is likely to be caused by increased rigidity in laryngeal and respiratory muscles and the associated inability to keep the laryngeal muscles in a fixed position.

#### 4.2.3. Analysis Methods

As depicted in Figure 4B, statistical analyses, where individual features are tested for significance, are described along with datasets for PD, speech impairments, stress, and depression. Lee Silverman Voice Treatment (LSVT) is usually assessed in such manner (63, 79), and studies describing novel ways in collecting datasets [e.g., (45)] rely on such statistical descriptions.

From the ‘traditional' predictive modeling approaches, support vector machines (SVMs) are most frequently used, which is in line with the baseline of the Interspeech ComParE challenge (16). Regression approaches are suitable to map disorder assessment scales (e.g., UPDRS for PD) but can potentially struggle with small sample sizes and unbalanced class distributions.

Approaches using neural networks are gaining popularity in recent years and are discussed in the following review (27). The recent ComParE 2018 and 2019 (19, 20) introduced features from deep representations as baseline methods in the domain of computational paralinguistics. This approach was pursued by Baird et al. (61) in the retrieved studies. In the other studies utilising neural networks, various network architectures are used. The way in which raw audio signals are processed and fed into neural networks depends strongly on the employed network architecture.

The overall goal of predictive modeling approaches is to create models that learn to generalise and therefore could classify voice samples of speakers, who were not present in the original dataset. To evaluate how well suggested predictive modeling approaches would perform at that task, the dataset should be split up into train, validation and test partitions. The train partition serves to adjust and fine-tune parameters of the model and those adjustments are then tested on the validation partition. The best performing model is then evaluated on the test partition, a hold-out part of the dataset (or ideally even a completely independent dataset with samples from the same disorder). This hold-out part should provide a sound judgement on how the model performs on data that it did not encounter during training/validation. Speakers have to be separated through all partitions since otherwise, the model can learn to identify a user and not learn the underlying information about the disease itself.

For imbalanced class distributions, which can be common in datasets with neurological disorders, the unweighted average recall (UAR) is the metric used in ComParE and should be used for comparing results across different predictive modeling approaches. Low et al. (17) provide some further, helpful advice for evaluation and validation of modeling approaches. Foremost, they advocate for using nested bootstrapping for a more robust performance estimation on small (< 100 patients) datasets. Ideally, the train, validation and test partitions would each represent the whole subject population of the dataset, but since this is unlikely for smaller subject numbers, nested bootstrapping provides a means to describe the mean or median estimate over a multitude of evaluation runs.

### 4.3. Emerging Trends

Some of the studies included in this review used a non-conventional clinical data recording setup. Those approaches can be categorized in a) data collection performed ‘in everyday life' and b) data collected from multiple modalities. Both categories are introduced further in the following section to provide an overview of these emerging trends.

#### 4.3.1. Everyday-Life Data Collection

Traditionally, medical datasets for analysing the impact of a disorder on voice were recorded in controlled recording conditions with relatively small sample sizes, since access to patients is a big obstacle to overcome and only possible through clinical institutions. Predictive modeling approaches and results from statistical analyses should be as general and flexible as possible, and also work on novel participants, who were not part of the initially recorded data. This requirement led to efforts in recent years to collect large-scale datasets. In those datasets, participants are often recruited not only at a clinic, but through interest groups and networks for disorders (80). Data collection itself is then being done remotely, in an offsite setup, through mobile devices such as smartphones (45) and smartwatches (55). These efforts are very promising to push the field toward a real-world use case, in which enough data can be collected to extrapolate models to work sufficiently well when confronted with completely novel data.

##### 4.3.1.1. Example Studies

In most clinical datasets, participants are only screened once since there is an increased effort to track and re-invite participants. Systems with which participants can provide several samples over a given observation time (49), are a big advantage and opportunity of large-scale data collection efforts. This can provide valuable insights in researching longitudinal disease courses [e.g., (48)], but recording sessions have to be designed differently than clinical sessions to put particular emphasis on adherence, therefore reducing user burden, and to motivate the user to record multiple times.

The overview in Figure 3 presenting which speech tasks are most commonly used in existing datasets, can provide some considerations on which speech tasks can be prioritized when user time is a considerable factor. Therefore, a legit approach could be to design a minimalist, user-friendly recording protocol, set up a small, clinical pre-study to validate that the relevant indications for the disease to be assessed are covered, and then use that minimalist protocol in a large scale data collection effort. According to our systematic screening, it depends on the disorder, but free speech and read speech tasks are most commonly used and could therefore make up a minimalist protocol.

After literature screening for this review, a publication was released, which showcases the highlighted points for everyday-life data collection (81). The authors managed to gather voice samples via a web app of over 6,650 participants, of which roughly 10 % reported to be depressed. They are piloting an extensive survey with 17 speech tasks, which on one hand seems to impair adherence (of 6,650, only 1,382 participants completed at least two of the total four survey versions), but on the other hand, can provide valuable insights into which speech tasks indeed carry most relevant information. This goes to show that a careful balance between user burden and the information to be collected is to be considered.

##### 4.3.1.2. Practical Considerations

The effort to bridge the gap between research and a real-world use case, however, is very high in the healthcare setup, since stakes are exceedingly more grave than in other fields. For example, providing an unsuitable product recommendation in an e-commerce setup is intuitively less detrimental than mislabelling a potential patient in a healthcare setup, where diagnosis or therapy decisions might be impacted. Therefore, even in large-scale data collection efforts, representing a whole population of potential later users is still a challenge, but a big step toward the right direction. Before generalising to everyday-life use cases, rigorous validation of experimental results is required, including quantification of changes in speech with time or treatment, as emphasised by Robin et al. (82).

Other challenges in large-scale data collections are non-standardised recording conditions. In controlled, clinical setups, high-quality microphones and even recording booths are used [e.g., (31)], but when collecting the data remotely from the user, microphone types might vary along with the variety of different smartphones on the market [e.g., (45)]. A few studies reported experiences and ideas to combat those issues [e.g., (83)]. Additionally, knowing beforehand which features are expected to be affected by the disorder to be studied can help when trying to adjust the data analysis pipeline respectively [e.g., (17)].

Obtaining reliable ground truth labels is another relevant aspect when participants are not recorded in a controlled clinical setup. Usually, participants are asked to self-annotate their data. To ensure a sufficient quality for these labels, it has to be ensured that participants can properly understand the applied labels themselves, and that the labeling process should be made as straightforward and effortless as possible (84).

A further consideration for large-scale data collection efforts is recruitment and user adherence. In clinical setups, cohorts are usually available through patients who are regularly treated in the clinic itself. If those patients are usually belonging to a rather elderly cohort (e.g., PD), specific considerations are required to ensure that smart devices to be used for large-scale data collection can be intuitively used and do not cause user frustration (72). To obtain data from a larger number of patients, the available cohort at a clinic might not be sufficient. Interest groups and networks for particular disorders can be a viable source to recruit patients (80), and healthy participants can be reached through online marketing or platforms such as Amazon Mechanical Turk [e.g., as done in R'mani Haulcy et al. (85)].

Another consideration and challenge for large-scale data collection is the identification of unique users. Machine learning systems in the voice analysis domain can easily overfit when no clear speaker separation is done. Since in anonymous data collection efforts [e.g., Zhang et al. (45)], it cannot be ruled out that the same speaker donates multiple samples, evaluation of the system's performance might be biased. Recruiting a clear set of speakers can be a solution, or using a setup in which the user has to register with a unique ID [e.g., via email address, Hecker et al. (86)].

##### 4.3.1.3. Data Privacy

A major and not negligible caveat in data collection approaches in everyday life is that the collected voice data might contain identity revealing aspects, and therefore, potential misuse could bear severe consequences. Especially in longitudinal data collection efforts, the longer the data collection effort continues, the more information from a patient is being collected, and the likelier a potential breach could be.

In a commercial setting, the technology of voice assistants seems promising at first glance to be utilised to quantify the status of disorders from voice. Voice assistants like Amazon Echo and Google Home are widespread and people interact readily with them through ‘free speech' prompts. Recently, some research has been done to find ways in which health-related processing of voice assistant queries can be implemented in a privacy-preserving way [e.g., (87, 88)]. However, privacy considerations on medical (voice) data collected in everyday life are a magnitude higher in the medical context than in private usage scenarios, and therefore, the technology is not yet widely used for medical voice collection yet (89). The majority of data collection efforts in everyday life identified within this review nevertheless focuses on dedicated implementations: custom apps on the smartphone (48, 49, 55, 72) and web sites (40, 45, 65). That way, data is not being processed or residing on the third party system of a voice assistant.

#### 4.3.2. Multiple Modalities

Another trend is the collection of data from multiple modalities. Predictive modeling approaches can gain performance when using more than a single modality, and this approach is known for some time already (90). In PD for example, gait is prominently affected besides voice (91). In affect-related disorders, such as major depression and bipolar disorder, video as an additional modality can carry complementary information on expressed emotion. The prominent Audio/Visual Emotion Challenge and Workshop (AVEC) addressed this aspect: featured sub-challenges in which audio and video data or features from clinical interviews (92) and interviews with virtual agents (93, 94) from the Distress Analysis Interview Corpus [DAIC, (32)] are provided as well as data on bipolar disorder (95). In addition, setups in which data is collected from the smartphone's camera as additional video input within a commercial setup are nowadays easily conceivable (96). The number of smart devices with sensors is constantly growing and therefore this topic has also been increasingly reflected in more recent dataset publications in this review (40, 48, 65, 72).

##### 4.3.2.1. Example Studies

The datasets we identified, which used multiple modalities, were recorded from voice data from patients with PD, stress, and ALS. Interestingly, apart from the traditional pairing of voice and video [as in Gratch et al. (32) for depression], some other modalities in combination with speech emerged. For PD, researchers used sensor data to additionally assess the motoric capabilities of the patients through a commonly used finger tapping task, a walking task, and a memory task (72). In another dataset, video, respiratory sinus arrhythmia, and heart rate data (36) were combined. Since PD affects motor coordination, assessing those modalities can yield some benefit, especially since (72) was done in a remote care setup.

Similarly, ALS affects muscle coordination and the studies using additional modalities to voice recorded physical activity and heart rate variability (65) as well as articulatory movement data (53, 64).

For stress, datasets were retrieved, which recorded biosignals such as blood volume pulse and skin conductance (61), as well as video data (38). Video data is frequently used to assist in the quantification of the expression of affect and therefore might also yield valuable additional information in a setting to elicit stress. Biosignals, such as skin conductance and blood volume pulse, are traditionally used to predict stress, and the attempt to infer them from the audio signal could pave the way to detect stress unobtrusively by voice only.

##### 4.3.2.2. Adapting the Data Processing Pipeline

When recording and analysing data collected from multiple modalities, however, the complexity of the recording setup and analysis pipeline is increased, since the different modalities need to be fused at some point in the analysis pipeline. If features are fused before predictive modeling algorithms are employed, the approach is termed ‘early fusion,' if multiple models for the respective modalities are created and their outputs are fused, it is termed ‘late fusion.'

In practice, increased complexity when conducting a study to record and analyse data as well as the need to still fully understand the effect of disorders on the voice modality are likely the reasons for focused datasets. But in line with the emerging trend toward everyday-life data collection, multimodal approaches could gain further popularity. When utilising participants' smartphones for data collection, their sensors already provide intrinsic additional modalities such as video, location, movement, and even device usage data. On the other hand, relying only on the voice modality could in practice lead to applications in settings where only that modality is available, for example when assessing phone calls (48).

### 4.4. Future Work

Based on the systematic screening of various original datasets from voice recordings of neurological disorders, we further highlight the following emerging trends. Future data collection endeavors will benefit prominently from collecting data in an everyday-life setup. Recording data in a clinical setup is a good means to explore specific nuances and aspects (e.g., symptoms) of a disorder further while recording data in everyday life enables insights into longitudinal disorder manifestation. Recording further modalities apart from audio can boost the performance of predictive modeling approaches. More research should be done on multi-modal data processing to balance the benefit of additional information and the cost of increased complexity.

## 5. Conclusion

To summarize, a variety of speech tasks are used in clinical practice, and usually, multiple tasks are recorded within one study to ensure that the relevant, distinct information for comparable analyses are covered. When regarding the common analysis methods utilised, we observe that custom feature extraction methods are quite prominent. However, established feature extraction toolkits within the research community yield the benefit of better comparability of the analysed features across different studies. Recently, learnt representations from deep learning toolkits are finding their way into the research community and offer an addition to the standard acoustic features.

The main contribution of this review is to provide a general overview of the field of neurological disorder recognition from voice. We emphasise how data collection efforts are undertaken, which trends emerge in the field, and aim to provide the readers with valuable practical insights. Lastly, we extend the overview of significantly correlating features for psychiatric disorders from Low et al. (17) and added prominent neurodegenerative disorders. This overview is particularly helpful when planning a data collection approach for a respective disorder to see which manifestations in voice are to be expected and to see with which speech task these could be captured.

## Data Availability Statement

The source code for generating the figures, including the raw tables of extracted data from the literature search as well as the extension of the figure of Low et al. (17) can be found on GitHub: https://github.com/Pascal-H/voice_analysis_for_neurological_disorder_recognition.

## Author Contributions

PH, FE, BS, and BA: conceptualisation. PH and NS: methodology (data identification and screening). PH, FE, and BA: analysis (syntheses). PH: writing – original draft preparation. PH, NS, FE, BS, and BA: writing – review and editing. All authors contributed to the article and approved the submitted version

## Funding

Funded by the Deutsche Forschungsgemeinschaft (DFG, German Research Foundation) - project number 491466077.

## Conflict of Interest

PH, FE, and BS were employed by the company audEERING GmbH. The remaining authors declare that the research was conducted in the absence of any commercial or financial relationships that could be construed as a potential conflict of interest.

## Publisher's Note

All claims expressed in this article are solely those of the authors and do not necessarily represent those of their affiliated organizations, or those of the publisher, the editors and the reviewers. Any product that may be evaluated in this article, or claim that may be made by its manufacturer, is not guaranteed or endorsed by the publisher.

## Footnotes

^1^ https://github.com/danielmlow/review/tree/389fc387a91f2d38004775ba7c94a970e3d1ae02

^2^ https://github.com/Pascal-H/speech_analysis_for_neurological_disease_recognition

## References

[1] Feigin VL Nichols E Alam T Bannick MS Beghi E Blake N . Global, regional, and national burden of neurological disorders, 1990-2016: a systematic analysis for the global burden of disease study 2016. Lancet Neurol. (2019) 18:459–80. 10.1016/S1474-4422(18)30499-X 30879893 PMC6459001

[2] Dorsey E Omberg L Waddell E Adams JL Adams R Ali MR . Deep phenotyping of Parkinson's disease. J Parkinsons Dis. (2020) 10:855–73. 10.3233/JPD-202006 32444562 PMC7458535

[3] Cummins N Scherer S Krajewski J Schnieder S Epps J Quatieri TF. A review of depression and suicide risk assessment using speech analysis. Speech Commun. (2015) 71:10–49. 10.1016/j.specom.2015.03.004

[4] Lu H Frauendorfer D Rabbi M Mast MS Chittaranjan GT Campbell AT . Stresssense: detecting stress in unconstrained acoustic environments using smartphones. In: Proceedings of the 2012 ACM Conference on Ubiquitous Computing. Pittsburgh, PA (2012). p. 351–60.

[5] Bourla A Mouchabac S El Hage W Ferreri F. e-PTSD: an overview on how new technologies can improve prediction and assessment of posttraumatic stress disorder (PTSD). Eur J Psychotraumatol. (2018) 9:1424448. 10.1080/20008198.2018.1424448 29441154 PMC5804808

[6] Parola A Simonsen A Bliksted V Fusaroli R. Voice patterns in schizophrenia: a systematic review and Bayesian meta-analysis. Schizophrenia Res. (2020) 216:24–40. 10.1016/j.schres.2019.11.031 31839552

[7] Van Puyvelde M Neyt X McGlone F Pattyn N. Voice stress analysis: a new framework for voice and effort in human performance. Front Psychol. (2018) 9:1994. 10.3389/fpsyg.2018.01994 30515113 PMC6255927

[8] Pulido MLB Hernández JBA Ballester MÁF González CMT Mekyska J Smékal Z. Alzheimer's disease and automatic speech analysis: a review. Expert systems with applications. (2020) 150:113213. 10.1016/j.eswa.2020.11321333833713

[9] Martínez-Nicolás I Llorente TE Martínez-Sánchez F Meilán JJG. Ten years of research on automatic voice and speech analysis of people with alzheimer's disease and mild cognitive impairment: a systematic review article. Front Psychol. (2021) 12:645. 10.3389/fpsyg.2021.620251 33833713 PMC8021952

[10] Chiaramonte R Bonfiglio M. Acoustic analysis of voice in bulbar amyotrophic lateral sclerosis: a systematic review and meta-analysis of studies. Logopedics Phoniatr Vocol. (2020) 45:151–63. 10.1080/14015439.2019.1687748 31760837

[11] Noffs G Perera T Kolbe SC Shanahan CJ Boonstra FM Evans A . What speech can tell us: a systematic review of dysarthria characteristics in Multiple Sclerosis. Autoimmunity Rev. (2018) 17:1202–9. 10.1016/j.autrev.2018.06.010 30316992

[12] Chiaramonte R Bonfiglio M. Acoustic analysis of voice in Parkinson's disease: a systematic review of voice disability and meta-analysis of studies. Revista de Neurologia. (2020) 70:393–405. 10.33588/rn.7011.2019414 32436206

[13] Patel RR Awan SN Barkmeier-Kraemer J Courey M Deliyski D Eadie T . Recommended protocols for instrumental assessment of voice: American speech-language-hearing association expert panel to develop a protocol for instrumental assessment of vocal function. Am J Speech Lang Pathol. (2018) 27:887–905. 10.1044/2018_AJSLP-17-0009 29955816

[14] Tóth L Hoffmann I Gosztolya G Vincze V Szatlóczki G Bánréti Z . A speech recognition-based solution for the automatic detection of mild cognitive impairment from spontaneous speech. Curr Alzheimer Res. (2018) 15:130–8. 10.2174/1567205014666171121114930 29165085 PMC5815089

[15] Eyben F Scherer KR Schuller BW Sundberg J André E Busso C . The Geneva minimalistic acoustic parameter set (GeMAPS) for voice research and affective computing. IEEE Trans Affect Comput. (2015) 7:190–202. 10.1109/TAFFC.2015.2457417

[16] Weninger F Eyben F Schuller BW Mortillaro M Scherer KR. On the acoustics of emotion in audio: what speech, music, and sound have in common. Front Psychol. (2013) 4:292. 10.3389/fpsyg.2013.00292 23750144 PMC3664314

[17] Low DM Bentley KH Ghosh SS. Automated assessment of psychiatric disorders using speech: a systematic review. Laryngosc Investigat Otolaryngol. (2020) 5:96–116. 10.31219/osf.io/5pwze 32128436 PMC7042657

[18] Horwitz R Quatieri TF Helfer BS Yu B Williamson JR Mundt J. On the relative importance of vocal source, system, and prosody in human depression. In: 2013 IEEE International Conference on Body Sensor Networks. Cambridge, MA: IEEE (2013). p. 1–6.

[19] Schuller B Steidl S Batliner A Marschik PB Baumeister H Dong F . The INTERSPEECH 2018. Computational paralinguistics challenge: atypical & self-assessed affect, crying & heart beats. In: Proceedings of Interspeech 2018. Hyderabad (2018). p. 122–6.

[20] Schuller BW Batliner A Bergler C Pokorny FB Krajewski J Cychosz M . The INTERSPEECH 2019. Computational paralinguistics challenge: styrian dialects, continuous sleepiness, baby sounds & orca activity. In: Proceedings of Interspeech 2019. (2019). p. 2378–82. 10.21437/Interspeech.2019-1122

[21] Boersma P Van Heuven V. Speak and unSpeak with PRAAT. Glot International. (2001) 5:341–347.

[22] Eyben F Weninger F Gross F Schuller B. Recent developments in opensmile, the munich open-source multimedia feature extractor. In: Proceedings of the 21st ACM International Conference on Multimedia. (2013). p. 835–8.

[23] Schmitt M Schuller B. Openxbow: introducing the passau open-source crossmodal bag-of-words toolkit. J Mach Learn Res. (2017) 18:3370–4. 10.48550/arXiv.1605.06778

[24] Amiriparian S Gerczuk M Ottl S Cummins N Freitag M Pugachevskiy S . Snore sound classification using image-based deep spectrum features. In: Proceedings of Interspeech 2017. (2017). p. 3512–6. 10.21437/Interspeech.2017-434

[25] Amiriparian S Freitag M Cummins N Schuller B. Sequence to Sequence autoencoders for unsupervised representation learning from audio. In: Proceedings of the Detection and Classification of Acoustic Scenes and Events 2017 Workshop (DCASE2017). Tampere: Tampere University of Technology, Laboratory of Signal Processing (2017). p. 17–21.

[26] Freitag M Amiriparian S Pugachevskiy S Cummins N Schuller B. audeep: unsupervised learning of representations from audio with deep recurrent neural networks. J Mach Learn Res. (2017) 18:6340–4. 10.48550/arXiv.1712.04382

[27] Cummins N Baird A Schuller BW. Speech analysis for health: current state-of-the-art and the increasing impact of deep learning. Methods. (2018) 151:41–54. 10.1016/j.ymeth.2018.07.007 30099083

[28] Voleti R Liss JM Berisha V. A review of automated speech and language features for assessment of cognitive and thought disorders. IEEE J Select Topics Signal Process. (2019) 14:282–98. 10.1109/JSTSP.2019.2952087 33907590 PMC8074691

[29] Page MJ McKenzie JE Bossuyt PM Boutron I Hoffmann TC Mulrow CD . The PRISMA 2020 statement: an updated guideline for reporting systematic reviews. Int J Surg. (2021) 88:105906. 10.1016/j.ijsu.2021.105906 33789826

[30] Gusenbauer M Haddaway NR. Which academic search systems are suitable for systematic reviews or meta-analyses? Evaluating retrieval qualities of Google Scholar, PubMed, and 26 other resources. Research Synthesis Methods. (2020) 11:181–217. 10.1002/jrsm.1378 31614060 PMC7079055

[31] Alghowinem S Goecke R Wagner M Epps J Breakspear M Parker G. Detecting depression: a comparison between spontaneous and read speech. In: 2013 IEEE International Conference on Acoustics, Speech and Signal Processing (ICASSP). Vancouver, BC: IEEE (2013). p. 7547–51.

[32] Gratch J Artstein R Lucas G Stratou G Scherer S Nazarian A . The distress analysis interview corpus of human and computer interviews. In: Proceedings of the Ninth International Conference on Language Resources and Evaluation (LREC'14). Reykjavik (2014). p. 3123–8.

[33] Jati A Williams PG Baucom B Georgiou P. Towards predicting physiology from speech during stressful conversations: heart rate and respiratory sinus arrhythmia. In: 2018 IEEE International Conference on Acoustics, Speech and Signal Processing (ICASSP). Calgary, AB: IEEE (2018). p. 4944–8.

[34] Knibb JA Woollams AM Hodges JR Patterson K. Making sense of progressive non-fluent aphasia: an analysis of conversational speech. Brain. (2009) 132:2734–46. 10.1093/brain/awp207 19696033 PMC2766235

[35] Weiner J Angrick M Umesh S Schultz T. Investigating the effect of audio duration on dementia detection using acoustic features. In: Proceedings of Interspeech 2018. Hyderabad (2018). p. 2324–8.

[36] Barnish MS Horton SM Butterfint ZR Clark AB Atkinson RA Deane KH. Speech and communication in Parkinson's disease: a cross-sectional exploratory study in the UK. BMJ Open. (2017) 7:e014642. 10.1136/bmjopen-2016-014642 28554918 PMC5730006

[37] Liu Z Li C Gao X Wang G Yang J. Ensemble-based depression detection in speech. In: 2017 IEEE International Conference on Bioinformatics and Biomedicine (BIBM). Kansas City, MO: IEEE (2017). p. 975–80. 10.1109/BIBM.2017.8217789

[38] Lefter I Burghouts GJ Rothkrantz LJ. An audio-visual dataset of human-human interactions in stressful situations. J Multimodal User Interfaces. (2014) 8:29–41. 10.1007/s12193-014-0150-7

[39] Fernandez R Picard RW. Modeling drivers' speech under stress. Speech Commun. (2003) 40:145–59. 10.1016/S0167-6393(02)00080-8

[40] Palacios-Alonso D Lázaro-Carrascosa C López-Arribas A Meléndez-Morales G Gómez-Rodellar A Loro-Álavez A . Assessing an application of spontaneous stressed speech-emotions portal. In: International Work-Conference on the Interplay Between Natural and Artificial Computation. Springer (2019). p. 149–60.

[41] Tsanas A Little M McSharry P Ramig L. Accurate telemonitoring of Parkinson's disease progression by non-invasive speech tests. Nat Prec. (2009) 57:884–93. 10.1038/npre.2009.3920.1 19932995

[42] Ikeno A Varadarajan V Patil S Hansen JH. UT-Scope: speech under lombard effect and cognitive stress. In: 2007 IEEE Aerospace Conference. Big Sky, MT: IEEE (2007). p. 1–7.

[43] Luz S. Longitudinal monitoring and detection of Alzheimer's type dementia from spontaneous speech data. In: 2017 IEEE 30th International Symposium on Computer-Based Medical Systems (CBMS). Thessaloniki: IEEE (2017). p. 45–6.

[44] Haider F De La Fuente S Luz S. An assessment of paralinguistic acoustic features for detection of Alzheimer's dementia in spontaneous speech. IEEE J Select Top Signal Process. (2019) 14:272–81. 10.1109/JSTSP.2019.2955022

[45] Zhang L Duvvuri R Chandra KK Nguyen T Ghomi RH. Automated voice biomarkers for depression symptoms using an online cross-sectional data collection initiative. Depression Anxiety. (2020) 37:657–69. 10.1002/da.23020 32383335

[46] Mendiratta A Scibelli F Esposito AM Capuano V Likforman-Sulem L Maldonato MN . Automatic detection of depressive states from speech. In: Multidisciplinary Approaches to Neural Computing. Cham: Springer (2018). p. 301–4.

[47] Rodríguez-Parra M Adrián J Casado J. Voice therapy used to test a basic protocol for multidimensional assessment of dysphonia. J Voice. (2009) 23:304–18. 10.1016/j.jvoice.2007.05.001 17658721

[48] Khorram S Jaiswal M Gideon J McInnis M Provost EM. The priori emotion dataset: linking mood to emotion detected in-the-wild. arXiv[Preprint].arXiv:180610658. (2018). 10.21437/Interspeech.2018-2355

[49] Maxhuni A Mu noz-Meléndez A Osmani V Perez H Mayora O Morales EF. Classification of bipolar disorder episodes based on analysis of voice and motor activity of patients. Pervasive Mobile Comput. (2016) 31:50–66. 10.1016/j.pmcj.2016.01.008

[50] Khan T Westin J Dougherty M. Classification of speech intelligibility in Parkinson's disease. Biocybernet Biomed Eng. (2014) 34:35–45. 10.1016/j.bbe.2013.10.003

[51] Sakar BE Isenkul ME Sakar CO Sertbas A Gurgen F Delil S . Collection and analysis of a Parkinson speech dataset with multiple types of sound recordings. IEEE J Biomed Health Inform. (2013) 17:828–34. 10.1109/JBHI.2013.2245674 25055311

[52] Sapir S Ramig LO Spielman JL Fox C. Formant centralization ratio: a proposal for a new acoustic measure of dysarthric speech. J Speech Lang Hear Res. (2010) 53:114–25. 10.1044/1092-4388(2009/08-0184) 19948755 PMC2821466

[53] Wang J Kothalkar PV Cao B Heitzman D. Towards automatic detection of amyotrophic lateral sclerosis from speech acoustic and articulatory samples. In: Proceedings of Interspeech 2016. San Francisco, CA (2016). p. 1195–9.

[54] Bose A van Lieshout P Square PA. Word frequency and bigram frequency effects on linguistic processing and speech motor performance in individuals with aphasia and normal speakers. J Neurolinguist. (2007) 20:65–88. 10.1016/j.jneuroling.2006.05.001

[55] Dubey H Goldberg JC Mankodiya K Mahler L. A multi-smartwatch system for assessing speech characteristics of people with dysarthria in group settings. In: 2015 17th International Conference on E-health Networking, Application & Services (HealthCom). Boston, MA: IEEE (2015). p. 528–33.

[56] An K Kim MJ Teplansky K Green JR Campbell TF Yunusova Y . Automatic early detection of amyotrophic lateral sclerosis from intelligible speech using convolutional neural networks. In: Proceedings of Interspeech 2018. Hyderabad (2018). p. 1913–7.

[57] Karan B Sahu SS Orozco-Arroyave JR Mahto K. Hilbert spectrum analysis for automatic detection and evaluation of Parkinson's speech. Biomed Signal Process Control. (2020) 61:102050. 10.1016/j.bspc.2020.102050

[58] Patel R. Acoustic characteristics of the question-statement contrast in severe dysarthria due to cerebral palsy. J Speech Lang Hear Res. (2003) 46:1401–15. 10.1044/1092-4388(2003/109) 14700364

[59] Galaz Z Mekyska J Mzourek Z Smekal Z Rektorova I Eliasova I . Prosodic analysis of neutral, stress-modified and rhymed speech in patients with Parkinson's disease. Comput Methods Progr Biomed. (2016) 127:301–17. 10.1016/j.cmpb.2015.12.011 26826900

[60] Orozco-Arroyave JR Arias-Londo no JD Vargas-Bonilla JF Gonzalez-Rátiva MC Nöth E. New spanish speech corpus database for the analysis of people suffering from Parkinson's disease. In: Proceedings of the Ninth International Conference on Language Resources and Evaluation (LREC'14). Reykjavik (2014). p. 342–7.

[61] Baird A Amiriparian S Berschneider M Schmitt M Schuller B. Predicting biological signals from speech: introducing a novel multimodal dataset and results. In: 2019 IEEE 21st International Workshop on Multimedia Signal Processing (MMSP). Kuala Lumpur: IEEE (2019). p. 1–5.

[62] Ho AK Iansek R Bradshaw JL. Motor instability in parkinsonian speech intensity. Cogn Behav Neurol. (2001) 14:109–16. 11417664

[63] Spielman J Ramig LO Mahler L Halpern A Gavin WJ. Effects of an extended version of the lee silverman voice treatment on voice and speech in Parkinson's disease. Am J Speech Lang Pathol. (2007) 16:95–107. 10.1044/1058-0360(2007/014) 17456888

[64] Kim Y Choi Y. A cross-language study of acoustic predictors of speech intelligibility in individuals with Parkinson's disease. J Speech, Lang Hear Res. (2017) 60:2506–18. 10.1044/2017_JSLHR-S-16-0121 28821018 PMC5831618

[65] Garcia-Gancedo L Kelly ML Lavrov A Parr J Hart R Marsden R . Objectively monitoring amyotrophic lateral sclerosis patient symptoms during clinical trials with sensors: observational study. JMIR mHealth uHealth. (2019) 7:e13433. 10.2196/13433 31859676 PMC6942190

[66] Chmielińska J Białek K Potulska-Chromik A Jakubowski J Majda-Zdancewicz E Nojszewska M . Multimodal data acquisition set for objective assessment of Parkinson's disease. In: Radioelectronic Systems Conference 2019, vol. 11442. International Society for Optics Photonics. Jachranka (2020). p. 114420F.

[67] Das B Daoudi K Klempir J Rusz J. Towards disease-specific speech markers for differential diagnosis in Parkinsonism. In: ICASSP 2019-2019 IEEE International Conference on Acoustics, Speech and Signal Processing (ICASSP). Brighton, UK: IEEE (2019). p. 5846–50.

[68] Altay EV Alatas B. Association analysis of Parkinson disease with vocal change characteristics using multi-objective metaheuristic optimization. Medical Hypotheses. (2020) 141:109722. 10.1016/j.mehy.2020.109722 32305812

[69] Tuncer T Dogan S Acharya UR. Automated detection of Parkinson's disease using minimum average maximum tree and singular value decomposition method with vowels. Biocybernet Biomed Eng. (2020) 40:211–220. 10.1016/j.bbe.2019.05.006

[70] Naranjo L Perez CJ Campos-Roca Y Martin J. Addressing voice recording replications for Parkinson's disease detection. Expert Syst Appl. (2016) 46:286–92. 10.1016/j.eswa.2015.10.034

[71] Smekal Z Mekyska J Galaz Z Mzourek Z Rektorova I Faundez-Zanuy M. Analysis of phonation in patients with Parkinson's disease using empirical mode decomposition. In: 2015 International Symposium on Signals, Circuits and Systems (ISSCS). Iasi: IEEE (2015). p. 1–4.

[72] Prince J Andreotti F De Vos M. Multi-source ensemble learning for the remote prediction of Parkinson's disease in the presence of source-wise missing data. IEEE Trans Biomed Eng. (2018) 66:1402–11. 10.1109/TBME.2018.2873252 30403615 PMC6487914

[73] Little M McSharry P Hunter E Spielman J Ramig L. Suitability of dysphonia measurements for telemonitoring of Parkinson's disease. Nat Prec. (2008) 56:1015. 10.1038/npre.2008.2298.1 21399744 PMC3051371

[74] Slegers A Filiou RP Montembeault M Brambati SM. Connected speech features from picture description in Alzheimer's disease: a systematic review. J Alzheimers Dis. (2018) 65:519–42. 10.3233/JAD-170881 30103314

[75] Mueller KD Hermann B Mecollari J Turkstra LS. Connected speech and language in mild cognitive impairment and Alzheimer's disease: a review of picture description tasks. J Clin Exp Neuropsychol. (2018) 40:917–39. 10.1080/13803395.2018.1446513 29669461 PMC6198327

[76] Clarke N Barrick TR Garrard P. A comparison of connected speech tasks for detecting early Alzheimer's disease and mild cognitive impairment using natural language processing and machine learning. Front Comput Sci. (2021) 3:634360. 10.3389/fcomp.2021.634360

[77] Bzdok D Meyer-Lindenberg A. Machine learning for precision psychiatry: opportunities and challenges. Biol Psychiatry. (2018) 3:223–30. 10.1016/j.bpsc.2017.11.007 29486863

[78] El Sharkawi A Ramig L Logemann J Pauloski BR Rademaker A Smith C . Swallowing and voice effects of Lee Silverman Voice Treatment (LSVT®): a pilot study. J Neurol Neurosurg Psychiatry. (2002) 72:31–6. 10.1136/jnnp.72.1.31 11784821 PMC1737706

[79] Sale P Castiglioni D De Pandis M Torti M Dallrmi V Radicati F . The Lee Silverman Voice Treatment (LSVT®) speech therapy in progressive supranuclear palsy. Eur J Phys Rehabil Med. (2015) 51:569–74. 26138088

[80] MacDonald B Jiang PP Cattiau J Heywood R Cave R Seaver K . Disordered speech data collection: lessons learned at 1 million utterances from project euphonia. In: Proceedings of Interspeech 2021. Brno (2021). p. 4833–7. 10.21437/Interspeech.2021-697

[81] Schwoebel JW Schwartz J Warrenburg L Brown R Awasthi A New A . A longitudinal normative dataset and protocol for speech and language biomarker research. medRxiv [Preprint]. (2021). 10.1101/2021.08.16.21262125

[82] Robin J Harrison JE Kaufman LD Rudzicz F Simpson W Yancheva M. Evaluation of speech-based digital biomarkers: review and recommendations. Digital Biomarkers. (2020) 4:99–108. 10.1159/000510820 33251474 PMC7670321

[83] Stasak B Epps J. Differential performance of automatic speech-based depression classification across smartphones. In: 2017 Seventh International Conference on Affective Computing and Intelligent Interaction Workshops and Demos (ACIIW). San Antonio, TX: IEEE (2017). p. 171–5.

[84] Yordanova K. Challenges providing ground truth for pervasive healthcare systems. IEEE Pervasive Comput. (2019) 18:100–4. 10.1109/MPRV.2019.2912261

[85] Ramani Haulcy JG. CLAC: a speech corpus of healthy English speakers. In: Proceedings of Interspeech 2021. (2021). p. 2966–70. d

[86] Hecker P Pokorny FB Bartl-Pokorny KD Reichel U Ren Z Hantke S . Speaking Corona? Human and machine recognition of COVID-19 from voice. In: Proceedings of Interspeech 2021. Brno (2021). p. 1029–33.

[87] Altuwaiyan T Hadian M Rubel S Liang X. Exploiting privacy-preserving voice query in healthcare-based voice assistant system. In: ICC 2020-2020 IEEE International Conference on Communications (ICC). Dublin: IEEE (2020). p. 1–6.

[88] Dojchinovski D Ilievski A Gusev M. Interactive home healthcare system with integrated voice assistant. In: 2019 42nd International Convention on Information and Communication Technology, Electronics and Microelectronics (MIPRO). Opatija: IEEE (2019). p. 284–8.

[89] Wienrich C Reitelbach C Carolus A. The trustworthiness of voice assistants in the context of healthcare investigating the effect of perceived expertise on the trustworthiness of voice assistants, providers, data receivers, and automatic speech recognition. Front Comput Sci. (2021) 53:685250. 10.3389/fcomp.2021.685250

[90] Fleury A Vacher M Noury N. SVM-based multimodal classification of activities of daily living in health smart homes: sensors, algorithms, and first experimental results. IEEE Trans Inf Technol Biomed. (2009) 14:274–83. 10.1109/TITB.2009.2037317 20007037

[91] Brognara L Palumbo P Grimm B Palmerini L. Assessing gait in Parkinson's disease using wearable motion sensors: a systematic review. Diseases. (2019) 7:18. 10.3390/diseases7010018 30764502 PMC6473911

[92] Valstar M Gratch J Schuller B Ringeval F Lalanne D Torres Torres M . Avec 2016: Depression, mood, and emotion recognition workshop and challenge. In: Proceedings of the 6th International Workshop on Audio/Visual Emotion Challenge. Amsterdam (2016). p. 3–10.

[93] Ringeval F Schuller B Valstar M Gratch J Cowie R Scherer S . Avec 2017: real-life depression, and affect recognition workshop and challenge. In: Proceedings of the 7th Annual Workshop on Audio/Visual Emotion Challenge. Mountain View, CA (2017). p. 3–9.

[94] Ringeval F Schuller B Valstar M Cummins N Cowie R Tavabi L . AVEC 2019 workshop and challenge: state-of-mind, detecting depression with AI, and cross-cultural affect recognition. In: Proceedings of the 9th International on Audio/Visual Emotion Challenge and Workshop. Nice (2019). p. 3–12.

[95] Ringeval F Schuller B Valstar M Cowie R Kaya H Schmitt M . AVEC 2018 workshop and challenge: Bipolar disorder and cross-cultural affect recognition. In: Proceedings of the 2018 on Audio/Visual Emotion Challenge and Workshop. Seoul (2018). p. 3–13.

[96] Neumann M Roesler O Liscombe J Kothare H Suendermann-Oeft D Pautler D . Investigating the utility of multimodal conversational technology and audiovisual analytic measures for the assessment and monitoring of amyotrophic lateral sclerosis at scale. arXiv[Preprint].arXiv:210407310. (2021). 10.21437/Interspeech.2021-1801

