# Supplementary material for: Voice Analysis for Neurological Disorder Recognition–A Systematic Review and Perspective on Emerging Trends
Source: Front Digit Health. 2022 Jul 7;4:842301. doi: 10.3389/fdgth.2022.842301 (PMC9309252; doi:10.3389/fdgth.2022.842301)
Supplement: Supplementary file 1 [file Table_1.pdf]

Supplementary Material: Neurological Disorder Recognition from Voice

These are the supplementary Tables S1 and S2 for the article 'Voice Analysis for Neurological Disorder Recognition – a Systematic Review and Perspective on Emerging Trends', https://doi.org/10.3389/fdgth.2022.842301.

1 SUPPLEMENTARY TABLES

Table S1: Identified datasets with multiple speech tasks.
Tasks: SV = Sustained vowels, RS = Read speech, DD = Diadochokinesis, FS = Free speech
*: Tasks which performed better than the other tasks within the same study.
Clinical assessments: UPDRS = Unified Parkinson's Disease Rating Scale, HY = Hoehn and Yahr scale, Webster = Webster Rating Scale for Parkinson's Disease, PHQ-9 = 9-question Patient Health Questionnaire, BDI = Beck's Depression Inventory, MINI = Mini-International Neuropsychiatric Interview, HAM-D = Hamilton Rating Scale for Depression.

	Subjects	Clinical assessment	Tasks	Notes
Parkinson's Disease (PD)				
(1)	20D<br>20 C	Clinical	**SV***, RS	"Vowels /e/, /i/ and /o/ are consistently the best along the classification experiments."
(2)	-	UPDRS suggested	SV, RS, DD	
(3)	25 D<br>0 C	Clinical: UPDRS	SV, RS, DD, FS	
(4)	20 D<br>20 C	Clinical: various	SV, RS	
(5)	98 D<br>51 C	Clinical: UPDRS	RS, **FS***	Poem recitation outperformed both reading tasks.
(6)	13 +<br>23 D	Clinical: UPDRS	SV, RS, FS	
(7)	50 D<br>50 C	Clinical: UPDRS, HY	SV, RS, DD, FS	"The results indicate that a selection of the speech features specific for a given speech task can in general increase prediction power of the regression model."

Table S1 continued from previous page.

	Subjects	Clinical assessment	Tasks	Notes
(8)	20 D<br>20 C	Clinical: UPDRS, HY	**SV***, RS	"[...] sustained vowels have been found to carry more PD-discriminative information than the isolated words and short sentences do."
(9)	42 D<br>0 C	Clinical UPDRS	SV, FS	"We used sustained vowels to avoid the confounding effects of running speech and thereby simplify the signal analysis."
(10)	12 D<br>0 C	Clinical: HY	SV, RS, FS	Tasks as different measures for the success of voice therapy and not classification performance.
(11)	13 D<br>13 C	Clinical: Webster	SV RS	"The extent of intensity decay was unchanged by the level of speech intensity for both speech tasks."
Depression				
(12)	222 D	Self: PHQ9	RS, **FS***	"The free speech task was better for predicting depression severity than the first task."
(13)	12 D<br>12 C	Clinical: BDI	RS, FS	
(14)	92 D<br>92 C	Clinical: MINI	RS, **FS***	The interview task has more diversity and gets higher accuracy than reading and picture description.
(15)	30 D<br>30 C	Self and clinical: HAM-D	SV, RS, **FS***	"[...] recognition rate using spontaneous speech was higher than for read speech."
Stress				
(16)	0 D<br>60 C		RS, FS	
Amyotrophic Lateral Sclerosis (ALS)				
(17)	25 D<br>0 C	Clinical: doctor	SV, RS	
(18)	11 D<br>11 C		SV, RS	
Aphasia, dysarthria and dysphonia				
(19)	21 D<br>21 C	Clinical: doctor	SV, RS, FS	

Table S2: Identified datasets with a single speech task.
SV = Sustained vowels, RS = Read speech, DD = Diadochokinesis, FS = Free speech
Clinical assessments: UPDRS = Unified Parkinson's Disease Rating Scale, HY = Hoehn and Yahr scale, PHQ-9 = 9-question Patient Health Questionnaire, STAI = State-Trait Anxiety Inventory, MMSE = Mini–Mental State Examination, Mesulam's criteria = Mesulam's criteria for primary progressive aphasia, HAM-D = Hamilton Rating Scale for Depression, ADS = Common Depression Scale, YRMS = Young Mania Rating Scale, MSS = Mania Self-Rating Scale.

	Subjects	Clinical assessment	Task
Parkinson's Disease (PD)			
(20), (21)	188 D<br>64 C	Clinical: doctor	SV
(22)	1513 D&C	Self: UPDRS (home)	SV
(23)	24 D<br>24 C	Clinical: doctor	RS
(24)	40 D<br>40 C	Clinical: doctor	SV
(25)	84 D<br>49 C	Clinical: doctor	SV
(26)	60 D<br>20 C	Clinical: UPDRS	RS
(27)	38 D<br>14 C	Clinical: HY	RS
(28)	23 D<br>8 C	Clinical: doctor	SV
Depression			
(29)	224 D<br>397 C	Self: i.a. PHQ-9	FS
Stress			

Table S2 continued from previous page.

	Subjects	Clinical assessment	Task
(30)	0 D<br>55 C	Self:<br>STAI	RS
(31)	0 D<br>32 C	Raters	FS
(32)	0 D<br>60 C	Physiological signals	FS
(33)	0 D<br>60 C	Raters	
(34)	0 D<br>4 C	Experiment condition	FS
**Alzheimer's Disease (AD)**			
(35)	82 D<br>82 C	Clinical:<br>MMSE, doctor	FS
(36)	71 D<br>268 C	Clinical:<br>doctor	FS
(37)	214 D<br>184 C	Clinical:<br>doctor	FS
**Amyotrophic Lateral Sclerosis (ALS)**			
(38)	13 D<br>13 C	Clinical:<br>doctor	RS
**Aphasia, dysarthria and dysphonia**			
(39)	15 D<br>15 C	Clinical:<br>Mesulam's criteria	FS
(40)	8 D<br>10 C	Clinical:<br>Doctor	RS
(41)	8 D<br>8 C	Clinical:<br>Intelligibility	RS
**Bipolar disorder**			
(42)	51 D<br>9 C	Clinical:<br>Assessment calls	FS

Table S2 continued from previous page.

	Subjects	Clinical assessment	Task
(43)	10 D<br>0 C	Clinical: HAM-D, ADS, YRMS, MSS<br>Self: Questionnaires	FS

REFERENCES

[1]Karan B, Sahu SS, Orozco-Arroyave JR, Mahto K. Hilbert spectrum analysis for automatic detection and evaluation of parkinson's speech. *Biomedical Signal Processing and Control* **61** (2020) 102050.

[2]Chmielińska J, Białek K, Potulska-Chromik A, Jakubowski J, Majda-Zdancewicz E, Nojszewska M, et al. Multimodal data acquisition set for objective assessment of parkinson's disease. *Radioelectronic Systems Conference 2019* (International Society for Optics and Photonics) (2020), vol. 11442, 114420F.

[3]Das B, Daoudi K, Klempir J, Rusz J. Towards disease-specific speech markers for differential diagnosis in parkinsonism. *ICASSP 2019-2019 IEEE International Conference on Acoustics, Speech and Signal Processing (ICASSP)* (IEEE) (2019), 5846–5850.

[4]Barnish MS, Horton SM, Butterfint ZR, Clark AB, Atkinson RA, Deane KH. Speech and communication in parkinson's disease: a cross-sectional exploratory study in the uk. *BMJ open* **7** (2017) e014642.

[5]Galaz Z, Mekyska J, Mzourek Z, Smekal Z, Rektorova I, Eliasova I, et al. Prosodic analysis of neutral, stress-modified and rhymed speech in patients with parkinson's disease. *Computer Methods and Programs in Biomedicine* **127** (2016) 301–317.

[6]Sale P, Castiglioni D, De Pandis M, Torti M, Dall'armi V, Radicati F, et al. The lee silverman voice treatment (lsvt®) speech therapy in progressive supranuclear palsy. *European Journal of Physical and Rehabilitation Medicine* **51** (2015) 569–74.

[7]Orozco-Arroyave JR, Arias-Londoño JD, Vargas-Bonilla JF, Gonzalez-Rátiva MC, Nöth E. New spanish speech corpus database for the analysis of people suffering from parkinson's disease. *Proceedings of the Ninth International Conference on Language Resources and Evaluation (LREC'14)* (2014), 342–347.

[8]Sakar BE, Isenkul ME, Sakar CO, Sertbas A, Gurgen F, Delil S, et al. Collection and analysis of a parkinson speech dataset with multiple types of sound recordings. *IEEE Journal of Biomedical and Health Informatics* **17** (2013) 828–834.

[9]Tsanas A, Little M, McSharry P, Ramig L. Accurate telemonitoring of parkinson's disease progression by non-invasive speech tests. *Nature Precedings* (2009) 1–1.

[10]Spielman J, Ramig LO, Mahler L, Halpern A, Gavin WJ. Effects of an extended version of the lee silverman voice treatment on voice and speech in parkinson's disease. *American Journal of Speech-Language Pathology* **16** (2007) 95–107.

[11]Ho AK, Iansek R, Bradshaw JL. Motor instability in parkinsonian speech intensity. *Cognitive and Behavioral Neurology* **14** (2001) 109–116.

[12]Zhang L, Duvvuri R, Chandra KK, Nguyen T, Ghomi RH. Automated voice biomarkers for depression symptoms using an online cross-sectional data collection initiative. *Depression and Anxiety* **37** (2020) 657–669.

[13]Mendiratta A, Scibelli F, Esposito AM, Capuano V, Likforman-Sulem L, Maldonato MN, et al. Automatic detection of depressive states from speech. *Multidisciplinary Approaches to Neural Computing* (Springer) (2018), 301–314.

[14]Liu Z, Li C, Gao X, Wang G, Yang J. Ensemble-based depression detection in speech. *2017 IEEE International Conference on Bioinformatics and Biomedicine (BIBM)* (IEEE) (2017), 975–980.

[15]Alghowinem S, Goecke R, Wagner M, Epps J, Breakspear M, Parker G. Detecting depression: a comparison between spontaneous and read speech. *2013 IEEE International Conference on Acoustics, Speech and Signal Processing (ICASSP)* (IEEE) (2013), 7547–7551.

[16]Ikeno A, Varadarajan V, Patil S, Hansen JH. Ut-scope: Speech under lombard effect and cognitive stress. *2007 IEEE Aerospace Conference* (IEEE) (2007), 1–7.

[17]Garcia-Gancedo L, Kelly ML, Lavrov A, Parr J, Hart R, Marsden R, et al. Objectively monitoring amyotrophic lateral sclerosis patient symptoms during clinical trials with sensors: observational study. *JMIR mHealth and uHealth* **7** (2019) e13433.

[18]Wang J, Kothalkar PV, Cao B, Heitzman D. Towards automatic detection of amyotrophic lateral sclerosis from speech acoustic and articulatory samples. *Proceedings of Interspeech 2016* (2016), 1195–1199.

[19]Rodríguez-Parra M, Adrián J, Casado J. Voice therapy used to test a basic protocol for multidimensional assessment of dysphonia. *Journal of Voice* **23** (2009) 304–318.

[20]Altay EV, Alatas B. Association analysis of parkinson disease with vocal change characteristics using multi-objective metaheuristic optimization. *Medical Hypotheses* **141** (2020) 109722.

[21]Tuncer T, Dogan S, Acharya UR. Automated detection of parkinson's disease using minimum average maximum tree and singular value decomposition method with vowels. *Biocybernetics and Biomedical Engineering* **40** (2020) 211–220.

[22]Prince J, Andreotti F, De Vos M. Multi-source ensemble learning for the remote prediction of parkinson's disease in the presence of source-wise missing data. *IEEE Transactions on Biomedical Engineering* **66** (2018) 1402–1411.

[23]Kim Y, Choi Y. A cross-language study of acoustic predictors of speech intelligibility in individuals with parkinson's disease. *Journal of Speech, Language, and Hearing Research* **60** (2017) 2506–2518.

[24]Naranjo L, Perez CJ, Campos-Roca Y, Martin J. Addressing voice recording replications for parkinson's disease detection. *Expert Systems with Applications* **46** (2016) 286–292.

[25]Smekal Z, Mekyska J, Galaz Z, Mzourek Z, Rektorova I, Faundez-Zanuy M. Analysis of phonation in patients with parkinson's disease using empirical mode decomposition. *2015 International Symposium on Signals, Circuits and Systems (ISSCS)* (IEEE) (2015), 1–4.

[26]Khan T, Westin J, Dougherty M. Classification of speech intelligibility in parkinson's disease. *Biocybernetics and Biomedical Engineering* **34** (2014) 35–45.

[27]Sapir S, Ramig LO, Spielman JL, Fox C. Formant centralization ratio: A proposal for a new acoustic measure of dysarthric speech. *Journal of Speech, Language, and Hearing Research* **53** (2010) 114–125.

[28]Little M, McSharry P, Hunter E, Spielman J, Ramig L. Suitability of dysphonia measurements for telemonitoring of parkinson's disease. *Nature Precedings* (2008) 1–1.

[29]Gratch J, Artstein R, Lucas G, Stratou G, Scherer S, Nazarian A, et al. The distress analysis interview corpus of human and computer interviews. *Proceedings of the Ninth International Conference on Language Resources and Evaluation (LREC'14)* (2014), 3123–3128.

[30]Baird A, Amiriparian S, Berschneider M, Schmitt M, Schuller B. Predicting biological signals from speech: Introducing a novel multimodal dataset and results. *2019 IEEE 21st International Workshop on Multimedia Signal Processing (MMSP)* (IEEE) (2019), 1–5.

[31]Palacios-Alonso D, Lázaro-Carrascosa C, López-Arribas A, Meléndez-Morales G, Gómez-Rodellar A, Loro-Álavez A, et al. Assessing an application of spontaneous stressed speech-emotions portal. *International Work-Conference on the Interplay Between Natural and Artificial Computation* (Springer) (2019), 149–160.

[32]Jati A, Williams PG, Baucom B, Georgiou P. Towards predicting physiology from speech during stressful conversations: Heart rate and respiratory sinus arrhythmia. *2018 IEEE International Conference on Acoustics, Speech and Signal Processing (ICASSP)* (IEEE) (2018), 4944–4948.

[33]Lefter I, Burghouts GJ, Rothkrantz LJ. An audio-visual dataset of human–human interactions in stressful situations. *Journal on Multimodal User Interfaces* **8** (2014) 29–41.

[34]Fernandez R, Picard RW. Modeling drivers' speech under stress. *Speech Communication* **40** (2003) 145–159.

[35]Haider F, De La Fuente S, Luz S. An assessment of paralinguistic acoustic features for detection of alzheimer's dementia in spontaneous speech. *IEEE Journal of Selected Topics in Signal Processing* **14** (2019) 272–281.

[36]Weiner J, Angrick M, Umesh S, Schultz T. Investigating the effect of audio duration on dementia detection using acoustic features. *Proceedings of Interspeech 2018* (2018), 2324–2328.

[37]Luz S. Longitudinal monitoring and detection of alzheimer's type dementia from spontaneous speech data. *2017 IEEE 30th International Symposium on Computer-Based Medical Systems (CBMS)* (IEEE) (2017), 45–46.

[38]An K, Kim MJ, Teplansky K, Green JR, Campbell TF, Yunusova Y, et al. Automatic early detection of amyotrophic lateral sclerosis from intelligible speech using convolutional neural networks. *Proceedings of Interspeech 2018* (2018), 1913–1917.

[39]Knibb JA, Woollams AM, Hodges JR, Patterson K. Making sense of progressive non-fluent aphasia: an analysis of conversational speech. *Brain* **132** (2009) 2734–2746.

[40]Bose A, van Lieshout P, Square PA. Word frequency and bigram frequency effects on linguistic processing and speech motor performance in individuals with aphasia and normal speakers. *Journal of Neurolinguistics* **20** (2007) 65–88.

[41]Patel R. Acoustic characteristics of the question-statement contrast in severe dysarthria due to cerebral palsy. *Journal of Speech, Language, and Hearing Research* **46** (2003) 1401–1415.

[42]Khorram S, Jaiswal M, Gideon J, McInnis M, Provost EM. The priori emotion dataset: Linking mood to emotion detected in-the-wild. *arXiv preprint arXiv:1806.10658* (2018).

[43]Maxhuni A, Muñoz-Meléndez A, Osmani V, Perez H, Mayora O, Morales EF. Classification of bipolar disorder episodes based on analysis of voice and motor activity of patients. *Pervasive and Mobile Computing* **31** (2016) 50–66.
